# Compositional and structural control in LLZO solid electrolytes†

**DOI:** 10.1039/d2ra03303h

**Published:** 2022-08-17

**Authors:** Kade Parascos, Joshua L. Watts, Jose A. Alarco, Yan Chen, Peter C. Talbot

**Affiliations:** National Battery Testing Centre, Queensland University of Technology Brisbane QLD 4001 Australia kade.parascos@hdr.qut.edu.au; Centre for Clean Energy Technologies and Practices, Centre for Materials Science, Queensland University of Technology Brisbane 4001 Australia; Neutron Scattering Division, Oak Ridge National Laboratory Oak Ridge TN 37831 USA

## Abstract

Garnet-based solid-state electrolytes (SSEs) represent a promising class of materials for next-generation batteries with improved safety and performance. However, lack of control over the composition and crystal structure of the well-known Li_7_La_3_Zr_2_O_12_ (LLZO) garnet material has led to poor reproducibility with a wide range of ionic conductivities reported in the literature. In this study, the role of precursor homogeneity in controlling the compositional and structural evolution of Al-doped LLZO is explored. A novel solution-based synthesis approach is employed to demonstrate enhanced atomic-scale mixing of the starting materials in comparison to conventional solid-state preparation methods. Through this technique, it is shown that the stability and formation temperature of the highly conductive cubic phase is directly impacted by the spatial distribution of the doping element and reactant species in the precursor mixture. Precursor homogeneity was also an important factor in mitigating the formation of unwanted secondary impurities. These findings can be used to guide the synthesis of SSEs with reproducible material characteristics and enhanced electrolytic performance.

## Abstract 2

Enhanced precursor homogeneity is achieved through a novel solution-based synthesis approach to enable precise control over material structure and composition.

## Graphical abstract

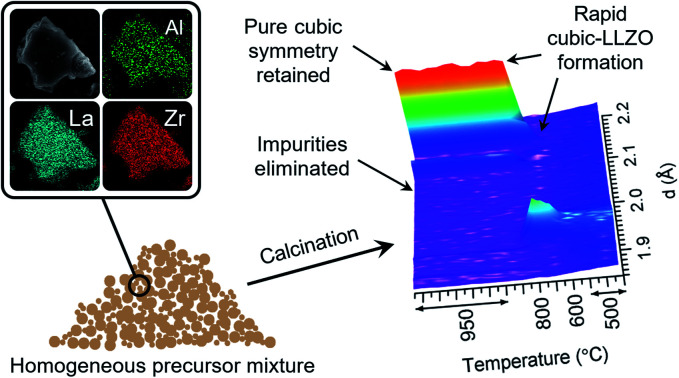

## Introduction

Over the past few decades, rechargeable lithium-ion batteries (LIBs) have emerged as essential energy storage devices for powering consumer electronics. In recent years, LIBs have found increasingly large-scale applications such as electric vehicles (EVs) and stationary energy storage systems (*i.e.*, electrical grid). However, critical safety issues still exist in the production and application of traditional LIBs due to the use of highly flammable organic solvents in the liquid electrolyte.^1^ These hazards are amplified in large-scale applications and several strategies have been proposed to mitigate such safety risks. One promising alternative to address the safety concerns in conventional LIBs is the use of all-solid-state battery (ASSB) cells, where the organic liquid electrolyte is replaced with a solid, often inorganic or polymeric electrolyte. The use of solid-state electrolytes may also allow increased energy densities by optimizing cell packaging and enabling the use of lithium metal anodes.^2,3^ Potential improvements in cycle life and stability are also motivating factors for the pursuit of advanced SSE materials.^4,5^

Among the SSE materials studied, the garnet LLZO structure has attracted great interest due to its high ionic conductivity (up to 1.6 mS cm^−1^ at room temperature) and unique stability against lithium metal.^6,7^ Two structural polymorphs are outlined in the literature with the first being the desired high-temperature cubic modification, which shows a disorder in the Li distribution and crystallizes in space group *Ia*3̄*d* (no 230).^8^ The second, a low-temperature tetragonal phase with a space group of *I*4_1_/*acd* (no 142), which exhibits complete ordering of Li-ions and an ionic conductivity two orders of magnitude lower than the cubic modification.^9^ Therefore, to avoid the formation of the tetragonal phase, supervalent dopants (*i.e.*, Al^3+^, Ga^3+^, Ta^5+^, Nb^5+^*etc.*) are substituted at the Li-, La- and Zr-sites to create Li vacancies and stabilise the cubic symmetry.^10–13^ Due to the superior conductivity of the cubic phase, control over the dopant stoichiometry and structural location in the garnet framework is critically important.

In addition to material crystal structure, the composition and microstructure are also important factors influencing the Li diffusion properties of LLZO electrolytes. A typical synthesis procedure for LLZO involves multiple heat treatments of metal oxides in excess of 1000 °C, making it difficult to maintain the control over the phase equilibrium and particle size of the product.^14^ Impurity phases such as La_2_Zr_2_O_7_, Li_2_ZrO_3_ and LaAlO_3_ commonly arise through conventional processing routes and are detrimental to the ionic conductivity.^15,16^ Various dopants can also participate in the synthesis reactions and introduce additional low-conductivity secondary phases. Li volatilization at high temperatures also complicates the synthesis process and introduces potential off-stoichiometry that can vary between batches.^17^ Lack of control over these variables results in poor reproducibility and contributes to the wide range of ionic conductivities and critical current densities (CCDs) reported in the literature. Recently, Weller *et al.* found that varying levels of elemental inhomogeneity in LLZO were largely responsible for the differences in reported performance properties.^18^ They revealed that inhomogeneities in the precursor mixture were mostly preserved even after high temperature treatment and were correlated with reduced ionic conductivity. However, by enhancing reagent mixing and using a narrow particle size distribution, the compositional homogeneity and, by extension, ionic conductivity of LLZO could be improved. These findings emphasize the importance of understanding and controlling the parameters that influence the composition and morphology of LLZO to optimize material performance.

In this work, *in situ* X-ray diffraction (XRD) and neutron powder diffraction (NPD) are used to investigate experimental factors influencing the chemical and structural evolution of LLZO during synthesis. In combination with scanning electron microscopy (SEM) and energy-dispersive spectroscopy (EDS) analysis, the impacts of precursor homogeneity on the phase purity and crystal structure of LLZO are explored. Focus is placed on the effects of the spatial distribution of the doping element on the stability of the desired cubic phase. To demonstrate enhanced control over material characteristics, a novel solution-based technique is employed to reproducibly synthesize phase-pure cubic-LLZO powders in a one-step calcination process. The synthetic approach enabled precise adjustment of the cation stoichiometry and ensured homogeneous substitution of the dopant species into the garnet structure. Samples were also synthesized by conventional solid-state processing to establish a benchmark for the comparison of material properties.

## Experimental details

### Solid-state synthesis

Al-doped LLZO (Al-LLZO) with a target composition of Li_6.28_Al_0.24_La_3_Zr_2_O_12_ was synthesized by solid-state (SS) reaction using stoichiometric amounts of LiOH·H_2_O (Sigma Aldrich, >98%), Al_2_O_3_ (Sumitomo Chemical, >99.99%), La(OH)_3_ (PI-KEM, >99.99%), and ZrO_2_ (PI-KEM, >99.5%). Nominal stoichiometry was selected based on neutron diffraction studies which show an Al doping level of 0.24 per formula unit (pfu) to optimize ionic conductivity while mitigating the formation of the low-conductivity tetragonal phase.^19^ Chemicals were weighed in a dry room (40 ppm H_2_O, −50 °C dew point) after being heated in a vacuum oven for 2 h at 80 °C to remove absorbed moisture. A 10 wt% excess of LiOH·H_2_O was used to compensate for Li volatilization at high temperatures. The precursors were combined inside a 100 mL zirconia milling jar with an equal mass of 2-propanol to assist with the milling process. Zirconia balls of 5 mm diameter were used as the milling media with a total ball mass of 125 g. The suspension was milled in a planetary ball mill (Changsha Deco Equipment Co, China) for 3 h with a rotational speed of 350 rpm. After milling, the balls were separated with a sieve and washed with 2-propanol to maximize recovery. The suspension was transferred to a glass tray and left overnight inside a fume hood to evaporate the solvent. The milled material was then pelletized in a 12 mm die with a press pressure of 1.5 tonnes for 2 minutes. Individual pellets were calcined in MgO crucibles at 950 °C for 5 h with a heating and cooling rate of 2.5 °C min^−1^ and 10 °C min^−1^, respectively. The calcination process was conducted in a preheated (150 °C) ceramic tube furnace (Labec, Australia) under air atmosphere with a gas flowrate of 0.5 L min^−1^. The calcined samples were transferred to a dry room at 250 °C upon cooling to minimize the interaction of LLZO with water vapour in ambient air. LLZO powders were uniaxially pressed and sintered in air at 1100 °C for 10 h inside an MgO crucible in a box furnace (Tetlow, Australia). The green pellets were covered with mother powder of the same composition to minimize Li loss during sintering.

### Solution-based synthesis

The solution-based (SB) synthesis route can be characterized as a modified sol–gel method. With a target composition of Li_6.28_Al_0.24_La_3_Zr_2_O_12_, stoichiometric amounts of LiNO_3_ (Suprapur, 99.995%), Al(NO_3_)_3_·9H_2_O (Novachem, 99.9%), La(NO_3_)_3_·6H_2_O (Thermo Fisher Scientific, 99.9%), and Zr(NO_3_)_2_(OH)_2_·4.7H_2_O (American Elements, 99%) were weighed in a dry room as they are all hygroscopic. Similar to the SS synthesis, a 10 wt% excess of LiNO_3_ was added to compensate for Li losses at high temperatures. Citric acid was used as the chelating agent with an amount equal to twice the total moles of cations in solution. The chelating agent was necessary to coordinate the metal cations in a citrate matrix to maintain homogeneity after evaporation and carbon removal. The Zr-hydroxynitrate precursor was first added to the citrate solution and stirred for 30 min at 40 °C. Due to the tendency of Zr-compounds to form insoluble zirconyl species in aqueous solution, H_2_O_2_ was added to oxidise Zr-ions and ensure the dissolution was complete and a colourless transparent solution was obtained. Following this, the remaining nitrate precursors were added to the solution and stirred for 1 h at 40 °C. The solution temperature was then raised to 80 °C to evaporate water until the stirrer bar seized upon the formation of a gel. The gel was transferred to an evaporator pot which was placed under vacuum for 30 min at 300 °C to remove the decomposed nitrates and residual moisture. The condensate was pelletized in a 12 mm die under a dry atmosphere at a pressure of 1.5 tonne for 2 minutes before being transferred to a ceramic tube furnace preheated to 150 °C. The calcination parameters and temperature profile for the SB sample were identical to the SS sample, however a 2 h isothermal dwell was incorporated on heating at 500 °C to fully decompose remaining organics and nitrates. Upon cooling, the sample was transferred to a dry room at 250 °C to reduce the interaction with ambient air. The sintering process used for LLZO powders prepared by the SB method followed the same methodology as the SS material.

### Characterisation

Morphology, composition, and structure of powder samples have been characterized using SEM, high-resolution transmission electron microscopy (HRTEM), EDS, thermogravimetric/-differential scanning calorimetry (TG/DSC), XRD, NPD, and inductively coupled plasma optical emission spectroscopy (ICP-OES). SEM micrographs were obtained on a JEOL JSM-7001 field emission instrument on Pt-coated powder samples mounted on an aluminium stub with carbon tape to enhance the conductivity of the specimen. HRTEM images were obtained on a JEOL JEM-2100 with an accelerating voltage of 200 kV on samples mounted on lacey carbon Cu-grids that were plasma-cleaned to minimize hydrocarbon contamination. TEM analysis was also performed on a JEOL JEM-ARM200F NEOARM with an automated aberration-correction system to obtain higher resolution images. For this instrument powder samples were mounted inside a titanium grid within a 50/50 mixture of araldite and carbon. After melting the epoxy/sample mixture into the grid at 120 °C, thin regions were prepared in the samples by mechanical polishing, dimple grinding and ion-milling.

Thermal analyses were conducted on a Netzsch Simultaneous Thermal Analyzer (STA) 449 F3 Jupiter in alumina crucibles with heating/cooling rates of 10 °C min^−1^ and 20 °C min^−1^, respectively. TGA and DSC signals were collected simultaneously with a constant air flowrate of 50 mL min^−1^.

Ambient XRD data was obtained on a Bruker D8 Advance Diffractometer using Co Kα_1_ radiation with an angle range from 0–90° and step size of 0.015°. Wide angle scans (15–160°) were also performed for refinement purposes. Phase identification was conducted in the DIFFRAC.EVA software using the 2022 Powder Diffraction File (PDF-4+) crystallographic database for peak indexing. Rietveld refinements of XRD data were performed in the Total Pattern Analysis Solutions (TOPAS) V6 software. Non-ambient XRD was performed on the precursor powders using a Rigaku Smartlab Diffractometer (Cu Kα_1_ radiation) equipped with an Anton Paar HTK 1200N oven chamber for *in situ* measurements. Samples were mounted in an alumina crucible and subject to the same temperature profile used during calcination with 15 min isothermal data collection points incorporated.

*In situ* NPD experiments were conducted at the VULCAN Engineering Materials Diffractometer,^20,21^ Spallation Neutron Source (SNS), Oak Ridge National Laboratory (Tennessee, USA). The green sample pellets (12 mm diameter) were stacked in a cylindrical MgO crucible (12.5 mm diameter) in order to increase sample volume and bulk density to allow for better statistics during neutron diffraction measurements. The sample was synthesized in the furnace following the same temperature profile used in calcination, and a positive flow of air through the furnace chamber was used. During the experiment, the high-flux mode and the 20 Hz chopper setting (0.5–3.5 Å d-space range at the 90° detector banks) were used, and the SNS was operated with the nominal 1.4 MW power. The 7 × 12 mm^2^ incident slit sizes and the 5 mm receiving collimators at the 90° detector banks defined the neutron gauge volume at the sample center, which excluded the backgrounds of the crucible and furnace. The time-of-flight neutron diffraction data were continuously collected throughout the synthesis and sliced with a time bin of 5 min using the VDRIVE software.^22^

Powders were prepared for ICP-OES analysis by digesting the material in 70 wt% HNO_3_. After digestion, the bulk composition was measured using an iCAP Pro X ICP-OES instrument and analyzed in the Qtegra V2.14 software.

## Results

A detailed analysis of precursors is provided, as control of the precursor stage is necessary to control the characteristics of the final material, as described below.

### Microstructure

**Fig. 1:**
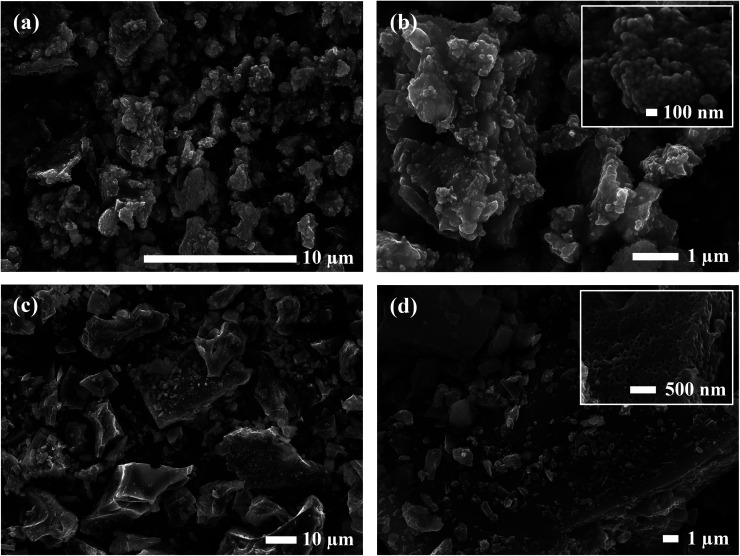
SEM micrographs of Al-LLZO precursor mixtures prepared by (a and b) SS and (c and d) SB synthesis methods.

SEM micrographs were taken to analyze the morphology of the materials before and after calcination. Fig. 1 shows the SEM images of the precursor powder mixtures at varying magnifications. Milled powders prepared by the SS approach exhibited irregular block-shaped secondary particles with a typical size of 3–7 μm. Closer inspection of the surface morphology revealed much smaller particulate units densely agglomerated with an approximate size of 100 nm, as shown in the inset of Fig. 1b. These distinct primary particles suggest that the precursor chemicals exist as individual crystalline particles closely packed together after milling. This is further evidenced by the sharp peaks in the *in situ* XRD diffraction pattern which were indexed to the as-received chemicals (see Fig. 10). In contrast, the condensate powder from the SB technique shows angular morphology with a non-uniform particle size distribution ranging from 100 nm to 20 μm. No distinguishable primary and secondary particles were observed, and the material appeared to show amorphous characteristics. This observation was also supported by the *in situ* XRD data showing a broad amorphous peak in the diffraction pattern of the starting material at room temperature (see Fig. 10). A microporous structure was also observed at higher magnifications (see inset of Fig. 1d) which is a resulting feature of the condensate formation process by rapid removal of residual H_2_O and nitrates.

**Fig. 2:**
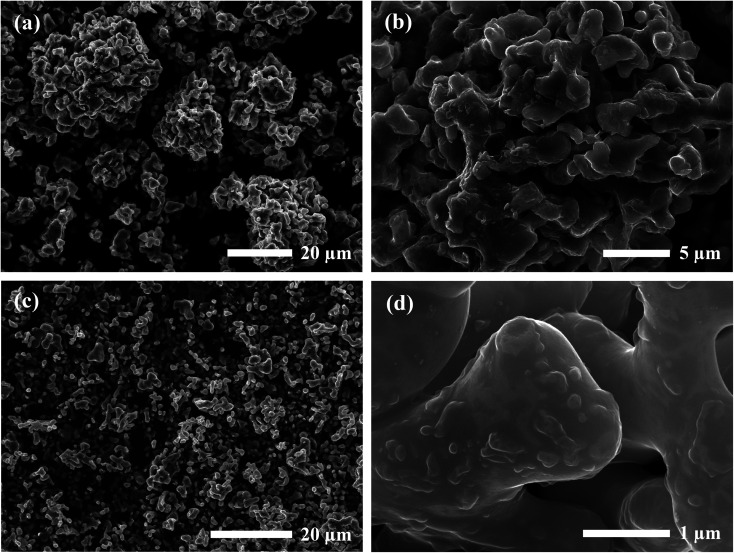
SEM images of calcined powders prepared by (a and b) SS and (c and d) SB processing.

SEM images of the calcined powders for each synthesis method are shown in Fig. 2. Samples prepared by the SB method showed a relatively uniform distribution of particles in the 1–2 μm size range. The rounded morphology of the particles indicates partial sintering of the material during calcination with evidence of necking found at higher magnifications. Remnants of smaller grains approximately 100 nm in diameter were also observed on the surface of the larger particles. Comparatively, the SS product formed a wider distribution of agglomerated particles ranging from 1–30 μm. These secondary particles were comprised of primary units 0.5–2 μm in diameter with irregular blocky morphology similar to that observed in the precursor material. Hence, the SS material exhibited a size factor approximately 10× greater than the SB sample for both primary and secondary particles.

**Fig. 3:**
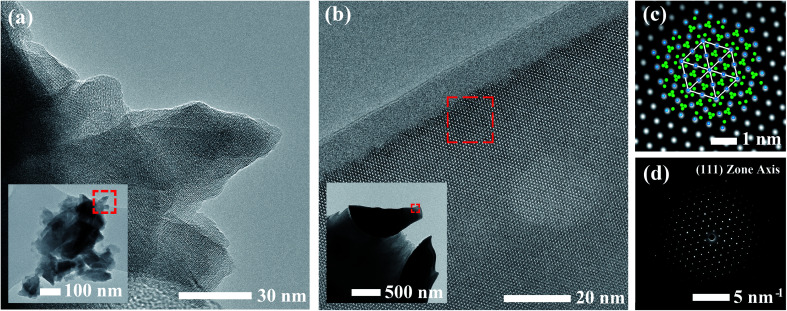
HRTEM images for Al-doped LLZO materials synthesized by (a) SS and (b) SB methods. The magnified region outlined by the red square in (b) is shown in the filtered image in (c) with the corresponding SAED pattern in (d). The (111) crystal configuration is superimposed onto (c) where Zr and La atoms are illustrated in blue and green, respectively.

Calcined materials were analyzed by HRTEM to obtain additional morphological information on a smaller length scale. Images of the SS sample obtained at high and low magnifications are shown in Fig. 3a. The inset of Fig. 3a reveals the irregular and multifaceted morphology of the SS particles consisting of multiple overlapping crystalline domains each with distinguishable orientations. The SS crystallites featured a broad size distribution ranging from 30–500 nm throughout the material.

HRTEM images for the SB material are shown in Fig. 3b. The material consisted of rounded, single crystalline entities 0.5–1 μm in diameter. In comparison to the SS sample, the SB sample exhibited more homogeneous character in terms of the size, shape, and degree of agglomeration of crystalline domains throughout the material. Due to the increased crystallite size in the SB material, powder samples were mounted on a titanium grid in a mixture of carbon/araldite and ion-milled to expose sufficiently thin regions for TEM analysis. The inset of Fig. 3b shows the edges of two ion-milled SB particles embedded in the carbon/araldite mixture. The magnified edge in Fig. 3b displays the strong diffraction pattern observed from the surface and extending into the depth of the particle. This crystal symmetry was indexed to the (111) zone axis in cubic LLZO. A filtered image of the magnified region is shown in Fig. 3c showing the recurring hexagonal pattern observed for the high-symmetry (111) zone axis with the selected area electron diffraction pattern (SAED) included in Fig. 3d. The crystal structure under this diffracting condition has been superimposed onto Fig. 3c with Zr and La atoms in blue and green, respectively. The 10–15 nm amorphous region at the edge of the particle is attributed to surface damage from ion-milling. The amorphous surface layer may also be partially due to carbonate/hydroxide formation caused by the interaction with ambient air.^23^

### Compositional analyses of precursors

**Fig. 4:**
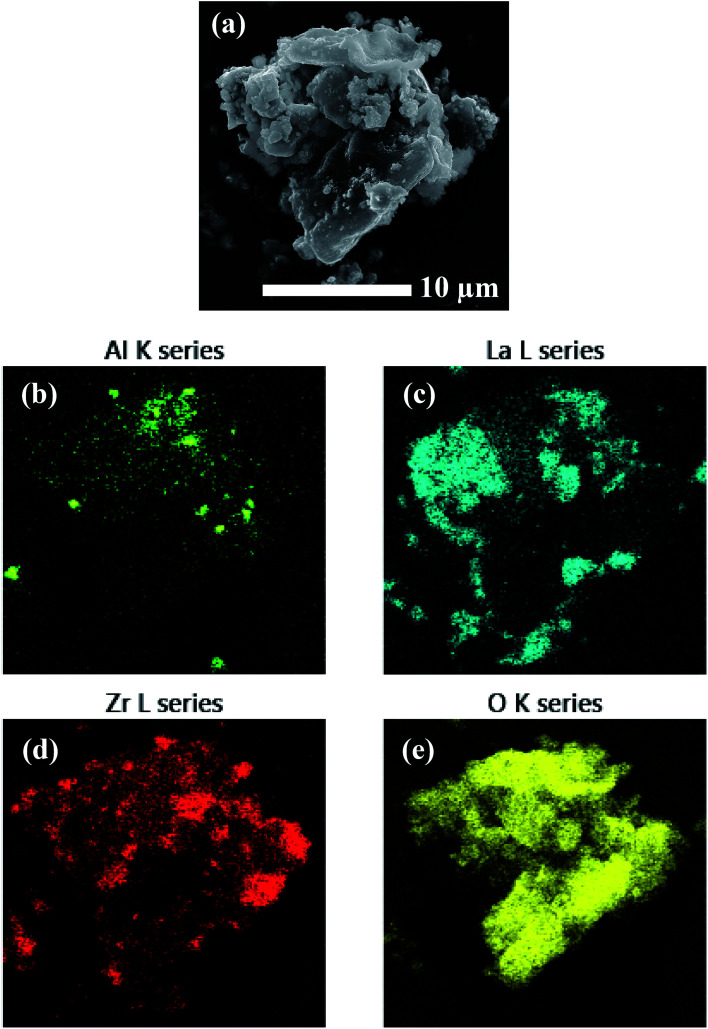
EDS mapping of a precursor particle prepared by SS processing showing (a) the electron micrograph and (b–e) the element map overlays.

**Fig. 5:**
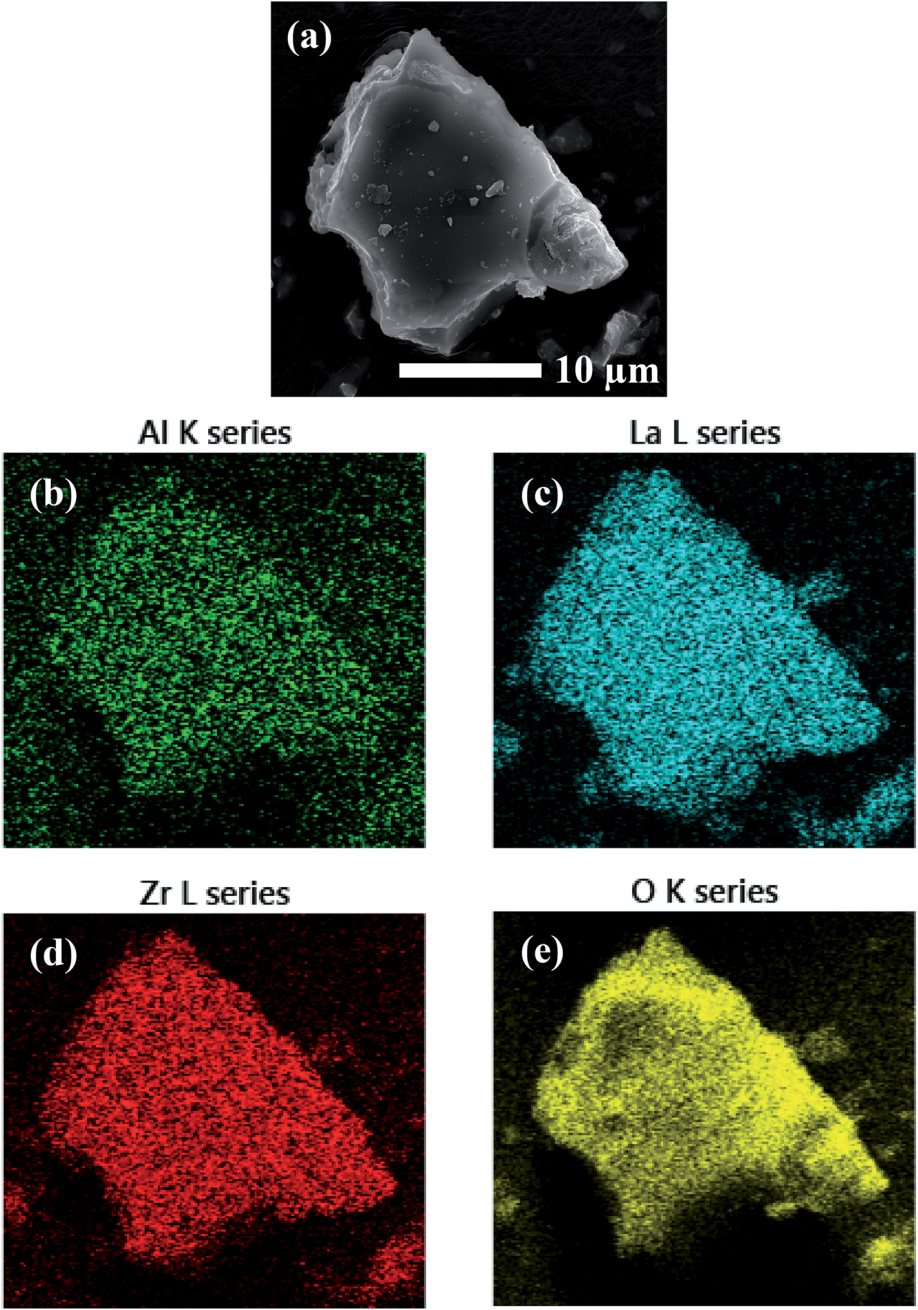
EDS mapping of a precursor particle prepared by SB processing showing (a) the electron micrograph and (b–e) the element map overlays.

EDS analysis was performed on the precursor mixtures to elucidate the differences in local elemental homogeneity between the preparation techniques. Fig. 4 shows an elemental map of a precursor particle prepared by the SS method with Al, La, Zr, and O distributions. The local distribution of cations is inhomogeneous with segregated clusters shown throughout the particle. Clustering of the Al distribution is clearly observed (see Fig. 4b). Fig. 5 shows the Al, La, Zr, and O series distributions for an isolated particle from the condensate powder prepared by the SB technique. The results demonstrate a highly homogeneous distribution of cations across the particle when compared with the SS sample, with no indication of segregated regions.

### Structural analyses of products

**Fig. 6:**
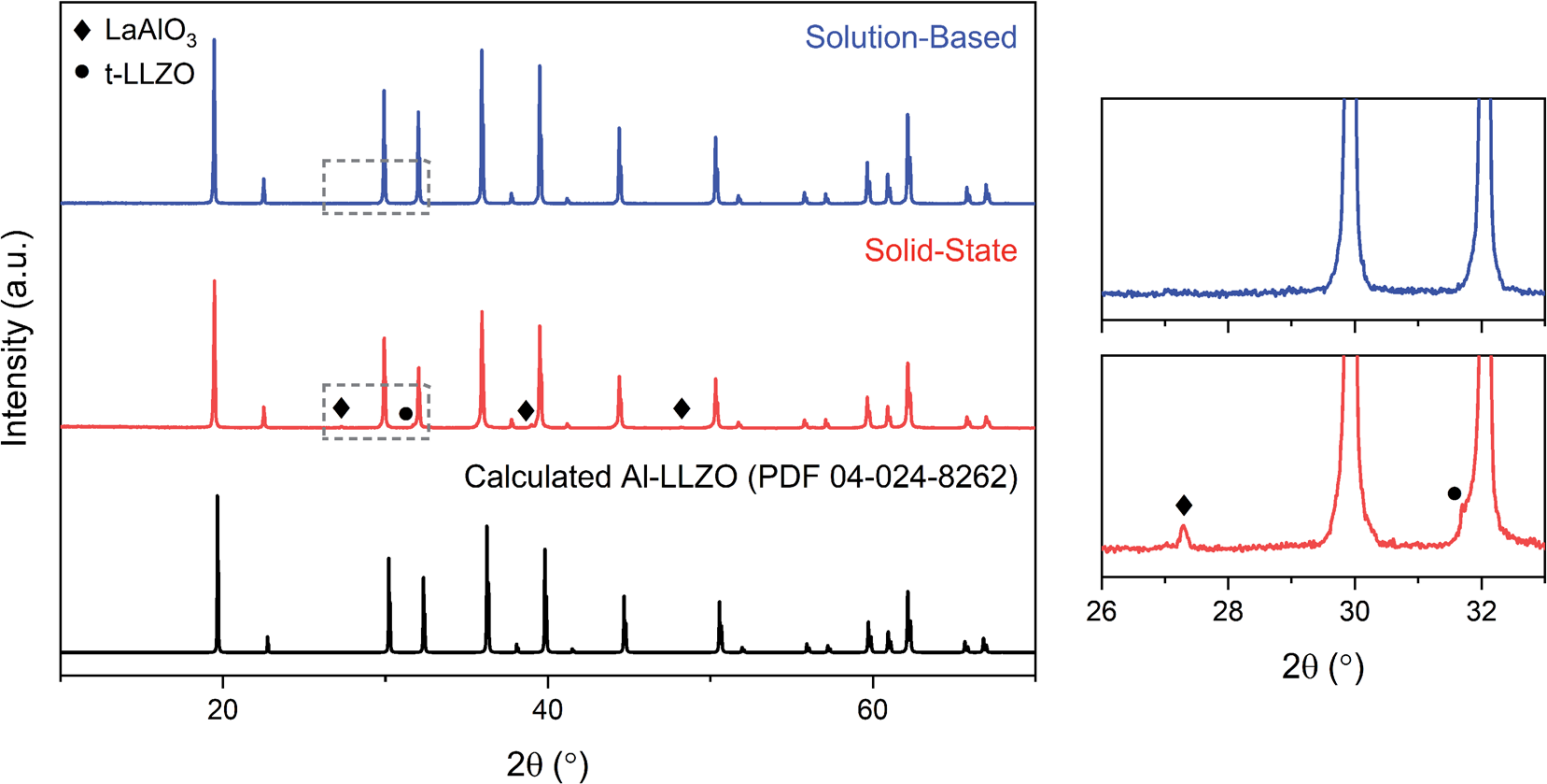
XRD patterns for calcined Al-LLZO powders prepared by the SB and SS synthesis methods. The theoretical diffraction pattern of cubic Al-LLZO is included for reference. At right is a magnified plot of the region outlined by the grey box.

The XRD patterns of the calcined powders are shown in Fig. 6. A phase-pure sample was produced by the SB method with no secondary phases found in the diffraction pattern. Comparatively, minor characteristic peaks indexed to the LaAlO_3_ impurity (PDF 04-006-0811) were detected in the SS sample as highlighted by the magnified range at right in Fig. 6. A shouldering effect was also observed in the SS material on several of the cubic peaks indicating the presence of tetragonal LLZO (PDF 01-080-6140). Another notable difference in the diffraction patterns was the reduction in peak intensity for the SS sample, with a 37% decrease for the most intense reflection at 2*θ* = 36° in comparison to the SB sample. Moreover, a peak broadening effect was observed for the SS sample with an increase in full width half maximum (FWHM) of 10–50% across all major peaks compared to the SB material. Statistical differences in peak intensity and width were consistent across the measured range of diffraction angles.

**Fig. 7:**
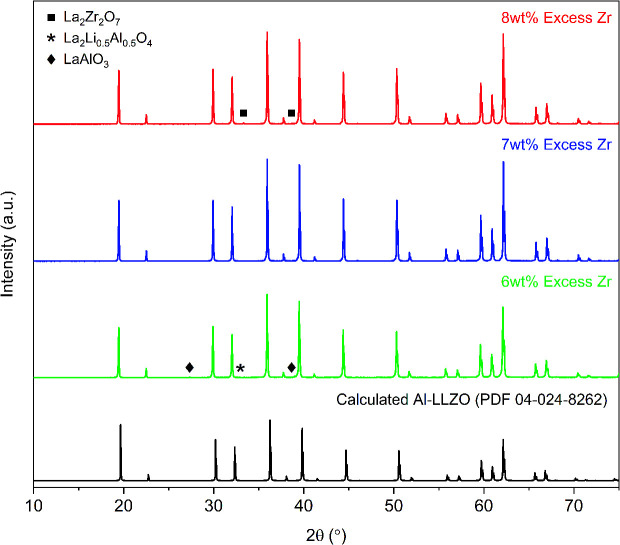
XRD patterns for calcined Al-LLZO powders with varying Zr excess prepared by the SB method. The theoretical diffraction pattern of cubic Al-LLZO is included for reference.

Initial diffraction patterns for the SB sample with stoichiometric additions of reagents showed strong peaks for the La_2_Li_0.5_Al_0.5_O_4_ intermediate phase (PDF 04-011-1108), indicating a potential Zr-deficiency in the system (see Fig. S1†). Since Zr-nitrate is known to sublime with a pressure of 26 Pa at 95 °C,^24^ it was likely that Zr-nitrate complexes in the gel were partially volatilized during the evaporation step leading to a Zr-deficiency in the condensate. This theory was tested by repeating the synthesis process with varying amounts of Zr-excess ranging from 5–10 wt%. The optimal range was found to be 6–8 wt% excess Zr and the diffraction patterns for each iteration are shown in Fig. 7. Trace amounts of the lanthanum aluminate impurities were detected at 6 wt% excess Zr indicating that a Zr deficiency was still present in the system. Conversely, an excess of 8 wt% led to the emergence of the La_2_Zr_2_O_7_ intermediate phase (PDF 01-080-6511), suggesting that the optimal Zr addition had been surpassed. Finally, a reproducible phase-pure product was obtained with a Zr-excess of 7 wt%. Hence, fine-tuning of the Zr-excess enabled precise control over the composition of the product.

To compare the sintering behavior of the materials, powders were uniaxially pressed and subjected to moderate sintering conditions (1100 °C, 10 hours) to form dense ceramic pellets. Note that this analysis is not focused on the optimization of the sintering process. Rather, it is intended to draw a preliminary comparison between the response of the materials to a typical sintering regime in the context of their initial homogeneity. Further systematic experimentation on the densification and electrochemical performance of the materials will be described elsewhere.

**Fig. 8:**
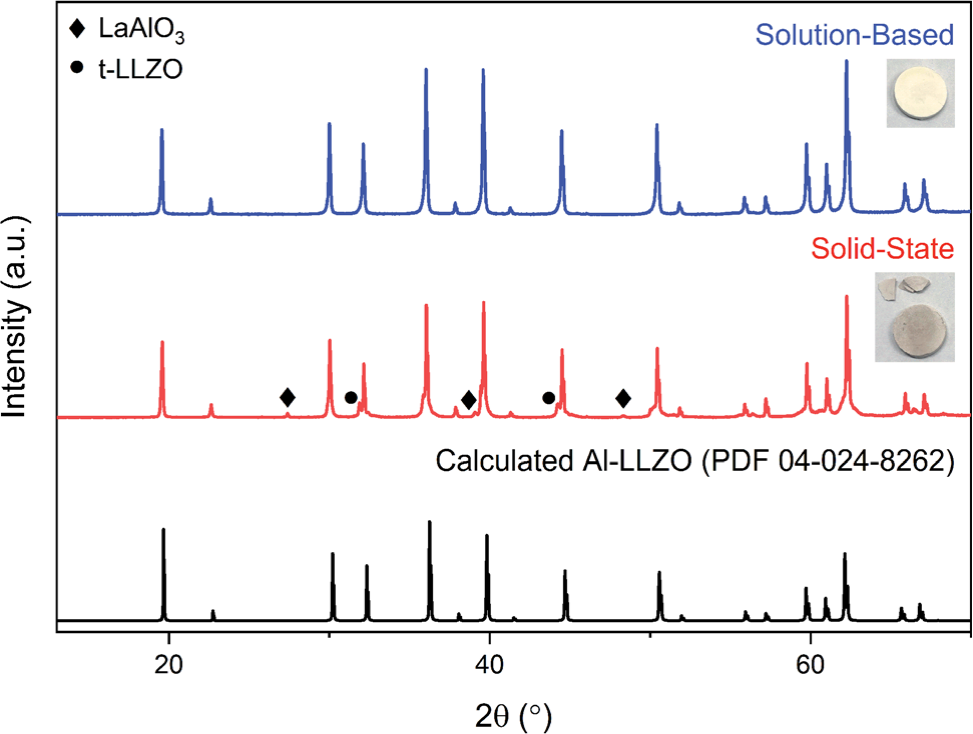
XRD patterns for sintered Al-LLZO pellets prepared by the SB and SS synthesis methods. The theoretical diffraction pattern of cubic Al-LLZO is included for reference.

Fig. 8 shows the diffraction patterns for the SS and SB pellet surfaces with photographs of each pellet included for visual comparison. An immediate distinction in the appearance of the pellets is the discoloration across the SS pellet which arises from phase inhomogeneity in the material. XRD patterns support this observation as impurity phases LaAlO_3_ and t-LLZO were detected in the material. Also, the SS material did not densify properly and remained in a powdered state after sintering, leading to cracking during handling as shown in the photograph. In contrast, the SB pellet exhibited uniform coloration and was consolidated into a dense ceramic body. The phase-purity of the SB material was also preserved after further heat treatment with no impurities identified in the diffraction pattern. Therefore, without incorporating additional grinding cycles or harsher sintering conditions, inhomogeneities and defects present in the SS calcined powders (see Fig. 6) were carried through to the final sintered pellet. In fact, the presence of the LaAlO_3_ phase was increased after densification due to the tendency of this phase to segregate on the surface and at grain boundaries.^16^ While mechanical polishing can partially remove some impurities from the surface, it was found that LaAlO_3_ was distributed throughout the bulk material and the diffraction pattern remained the same after polishing. Interestingly, there was no formation of lanthanum aluminate impurities on the SB pellet surface after sintering.

### Thermal analysis

**Fig. 9:**
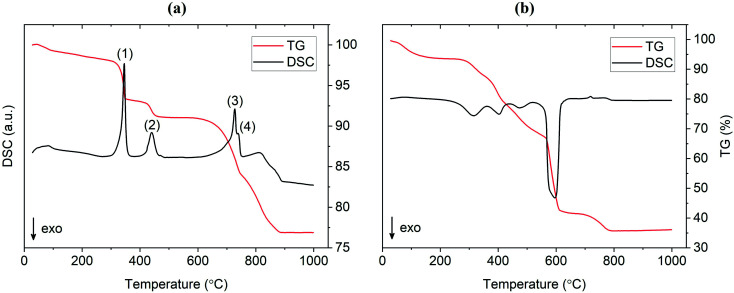
TG/DSC curves for Al-LLZO prepared by the (a) SS and (b) SB technique.

**Fig. 10:**
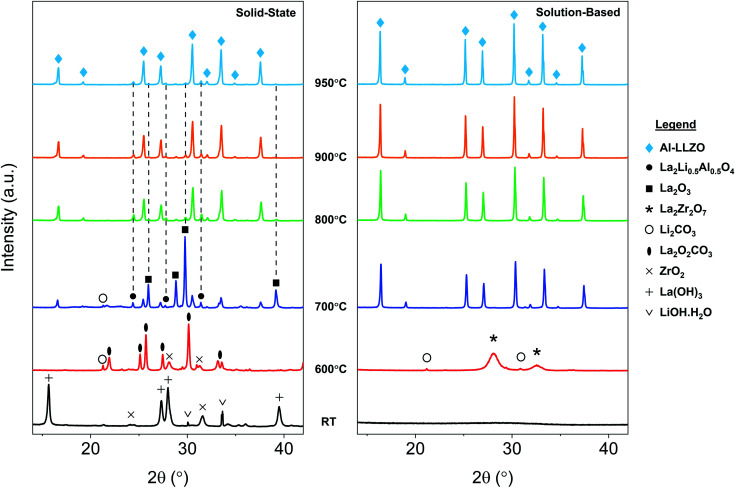
*In situ* XRD diffraction patterns for SS and SB samples upon heating during calcination. Grey dotted lines shown in the SS sample indicate residual phases at 950 °C.

TG/DSC experiments were conducted to analyze the thermal characteristics of the precursor mixtures. Fig. 9a shows the TG/DSC curves for the mixture prepared by SS reaction. The endothermic peaks (1) and (2) represent the dehydration reactions of the La(OH)_3_ precursor. This occurs by a two-step mechanism where La(OH)_3_ is first converted at 350 °C to the intermediate LaO(OH), which then further dehydrates at 450 °C to form La_2_O_3_. Each reaction is accompanied by a mass loss of 5% and 2%, respectively, as shown by the TG curve in red. Peak (3) at approximately 720 °C is attributed to the melting of Li_2_CO_3_ which is in agreement with the value from literature.^25^ While LiOH·H_2_O was used as the Li precursor, it is assumed that a significant portion of this compound would be converted to Li_2_CO_3_ since the reaction is carried out in air. This assumption is verified by the presence of characteristic Li_2_CO_3_ peaks in the diffraction pattern at 600 °C and 700 °C from *in situ* XRD measurements (see Fig. 10). The small endotherm at 760 °C (peak (4)) is assigned to the decomposition of lanthanum oxycarbonate, La_2_O_2_CO_3_, which is also formed by the reaction with carbonates in the system that arise from interactions with the atmosphere. The associated mass loss for peaks (3) and (4) are a result of CO_2_ evolution and Li_2_O vaporization, the latter being greatly accelerated by the presence of liquid Li_2_CO_3_. Overall, the sample experienced a total mass loss of approximately 23 wt% upon heating to 1000 °C.

TG/DSC curves for the material prepared by the SB method are shown in Fig. 9b. An immediate distinction to the SS material is the total mass loss during calcination, which was approximately as 64 wt% for the SB sample. This result was expected due to the large amount of organic material (citrates, nitrates *etc.*) that is vaporized during high temperature treatment. In addition, the large exotherm observed at 600 °C may be estimated as the activation point for crystallization as the weight change is relatively minimal past this temperature (∼5 wt%). This indicates that the onset of the formation of the cubic garnet starts around 600 °C, more than 100 °C less than values previously reported in the literature (typical phase onset from 710–720 °C).^17^*In situ* XRD data supports this result in showing a change from a broad amorphous to a sharp crystalline pattern from 600 °C to 700 °C. These findings suggest that the SB technique allows the synthesis of Al-LLZO at lower temperatures and shorter processing times.

### Phase evolution

*In situ* XRD was conducted to observe the phase evolution and reaction mechanism during the synthesis of Al-LLZO for each preparation method. Fig. 10 shows the diffraction patterns upon heating to 950 °C during calcination for the SS (left) and SB (right) sample. Residual impurities in the SS sample at 950 °C have been indicated by grey dotted lines following their formation at approximately 700 °C. It should be noted that the resulting phases from the *in situ* measurements differed slightly from the ambient XRD data due to changes in the furnacing equipment and sample geometry. Nonetheless, a range of critical distinctions between the synthesis methods were highlighted by the *in situ* analysis. Firstly, a clear contrast between the samples was the shape of the diffraction patterns of the starting precursor mixtures at room temperature (RT). For the SS sample, sharp crystalline peaks were observed for the starting chemicals LiOH·H_2_O, La(OH)_3_ and ZrO_2_ (Al_2_O_3_ content was below the detection limit for the measurement). These starting materials are reformed at 600 °C with peaks for La_2_O_2_CO_3_, ZrO_2_, and Li_2_CO_3_ identified in the measurement. Such transitions are accompanied by large enthalpy changes and were therefore detected in the TG/DSC analysis (see Fig. 9). In contrast, a broad amorphous peak was detected for the SB precursor at RT which appeared as a near-straight background line in Fig. 10. It was not until 700 °C that the material was fully crystallized with intermediate phases Li_2_CO_3_ and La_2_Zr_2_O_7_ appearing at 600 °C. The crystalline and amorphous nature of the precursor mixtures from the XRD data is supported by SEM observations which found separate crystalline entities with distinct primary particles in the SS precursor compared to homogeneous amorphous material in the SB precursor.

Another important distinction between the samples was the onset temperature of the formation of the cubic Al-LLZO phase. At 700 °C, the SS sample exhibited weak peaks for the Al-LLZO phase with strong reflections for La_2_O_3_ and La_2_Li_0.5_Al_0.5_O_4_, several of which remained present up until the final temperature of 950 °C. Comparatively, the Al-LLZO phase was fully formed in the SB sample by 700 °C and remained stable to 950 °C, suggesting that a phase-pure material could be obtained at lower processing temperatures and shorter dwell times.

**Fig. 11:**
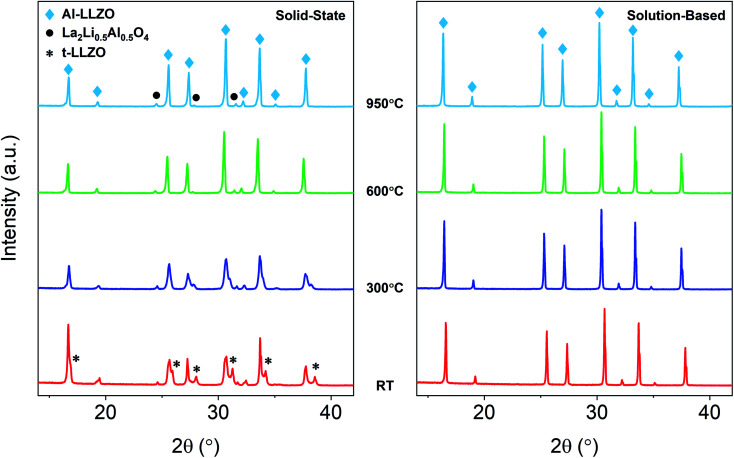
*In situ* XRD diffraction patterns for SS and SB samples upon cooling during calcination.

A detailed analysis of the phase transformations upon cooling is equally as important for the synthesis of Al-LLZO. This is because the cubic-to-tetragonal transition occurs during cooling, beginning from 600 °C and continuing down to RT.^17^ Diffraction patterns from the *in situ* experiment during cooling are shown in Fig. 11 with measurements taken at 600 °C, 300 °C and RT. The SS sample showed a significant destabilization of cubic Al-LLZO to the tetragonal modification with strong t-LLZO peaks evident at RT. In comparison, the SB sample was found to remain stable during cooling to RT with no reflections detected for the tetragonal phase. In accordance with ambient XRD measurements, peak intensities for cubic Al-LLZO were significantly reduced in the SS sample even at 950 °C, before any t-LLZO contributions could reduce the intensity of the cubic peaks.

**Fig. 12:**
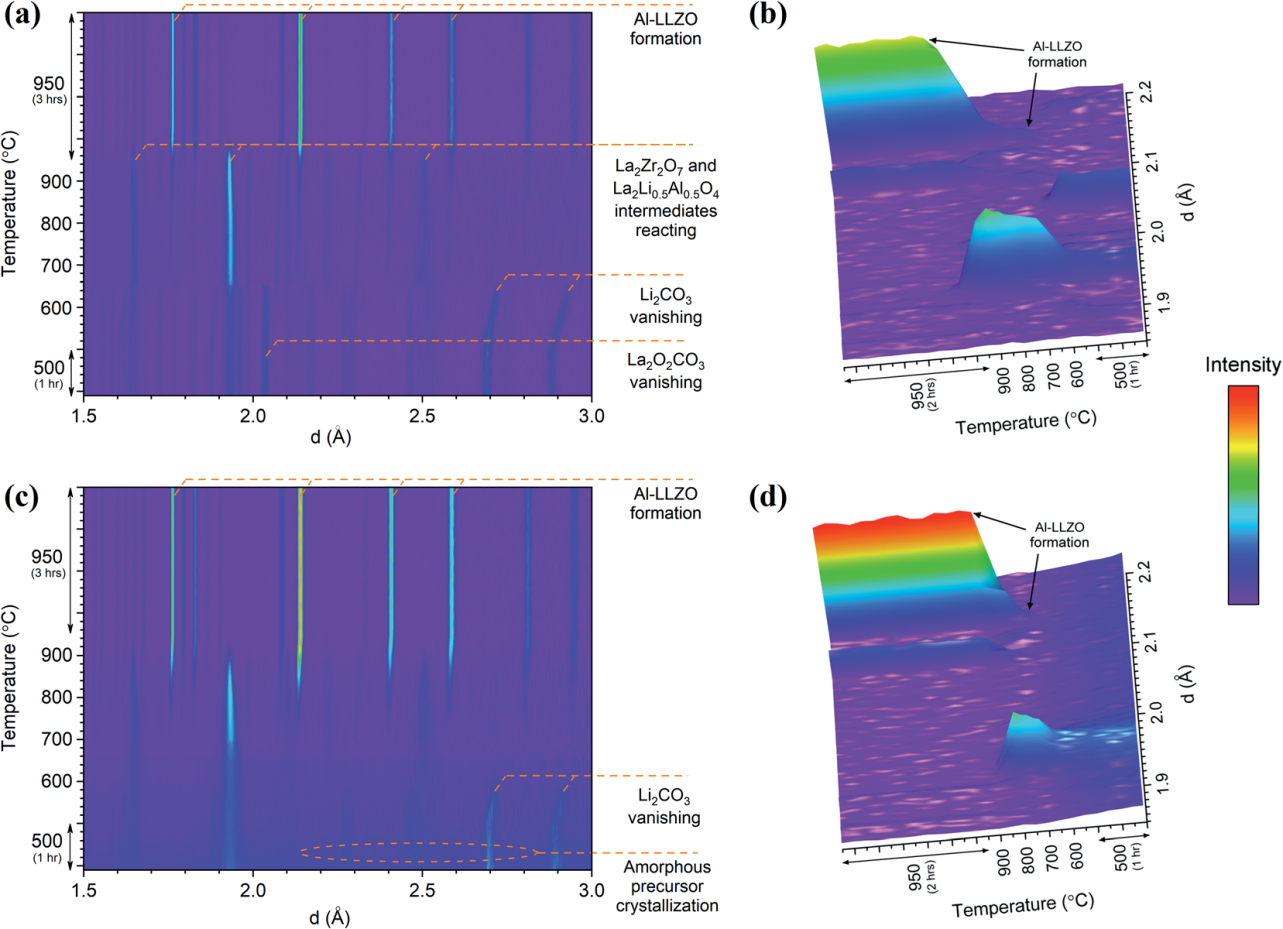
*In situ* NPD 2D and 3D surface plots for SS (a and b) and SB (c and d) samples during heating. The intensity scale has been applied equally across all plots.

*In situ* NPD experiments visualized the continuous phase evolution and revealed the synthesis mechanism of LLZO. In contrast to the XRD that collects the signal from the superficial sample layer, the high-resolution and deep-penetrating neutron diffraction captures the phase evolution information from the sample internal with bulk average. In addition, NPD possesses the unique sensitivities to light-weight elements to monitor the reactions of some precursors such as Li_2_CO_3_ that have weak signals in XRD. Detailed structural refinements with additional *ex situ* NPD experiments at RT are currently under way and will be presented elsewhere. Therefore, this analysis is treated qualitatively to highlight differences in the phase transformations between the alternate synthesis methods. Two- and three-dimensional (2D and 3D) surface plots for the SS and SB samples are shown in Fig. 12. The surface plots have been truncated to focus on the formation temperature region of cubic LLZO during heating. An immediate distinction between the two samples is the relatively crystalline nature of the material before transitioning to cubic LLZO in the SS sample in comparison to the SB sample, which exhibits broader peaks with greater background noise for the reactant material. This aligns with *in situ* XRD results showing characteristically broad amorphous peaks for the SB sample at 600 °C before rapidly transitioning to the crystalline Al-LLZO phase. In further agreement with XRD measurements, an increased intensity of the Al-LLZO peaks in the SB sample was also observed in the NPD analysis owing to the greater crystallinity and enhanced phase homogeneity of the solution-processed material. The difference in peak intensity is clearly illustrated in the 3D surface plots in Fig. 12b and d, which are both scaled to the same intensity range. Further details on the implications of diffraction peak profiles are included in the discussion.

The SS sample shows a gradual introduction of the cubic LLZO phase beginning at 710–720 °C and reaching its maximum phase fraction approximately 1 hour into the final dwell at 950 °C. This onset formation temperature agrees with the *in situ* NPD findings presented by Chen *et al.*^17^ In comparison to the SS sample, the SB sample exhibits a more rapid formation of Al-LLZO beginning at the same onset temperature and completing at 920 °C. The formation process is visualized by the 3D plots in Fig. 12b and d which focus on the most intense reflection of the Al-LLZO phase at ∼2.14 Å.

Another notable feature revealed by the NPD analysis is the difference in temperature at which Li_2_CO_3_ peaks vanish between the samples. For the SS sample, Bragg peaks for crystalline Li_2_CO_3_ disappeared at 660 °C in comparison to 580 °C for the SB sample. Above this temperature, broad background features appear in the SS surface plot due to the presence of a liquid carbonate melt. This effect is not as obvious in the SB sample since the starting material is already highly amorphous. Localized exothermic reactions (as detected by DSC) coupled with greater reactant homogeneity in the SB sample are likely responsible for the low-temperature disappearance of Li_2_CO_3_ reflections from melting. Since the garnet formation is accompanied by the melting and reaction of liquid Li_2_CO_3_,^17^ early vanishing of the carbonate peaks suggests that the final phase can be formed at lower temperature. However, due to surface *vs.* bulk effects described previously, the formation onset of the cubic garnet was delayed, and this result was not entirely reflected by the NPD measurements. Nonetheless, TG/DSC and *in situ* XRD results clearly support the notion that the garnet reaction onset temperature is reduced in the SB material.

### Evidence of Al incorporation into the cubic structure

**Fig. 13:**
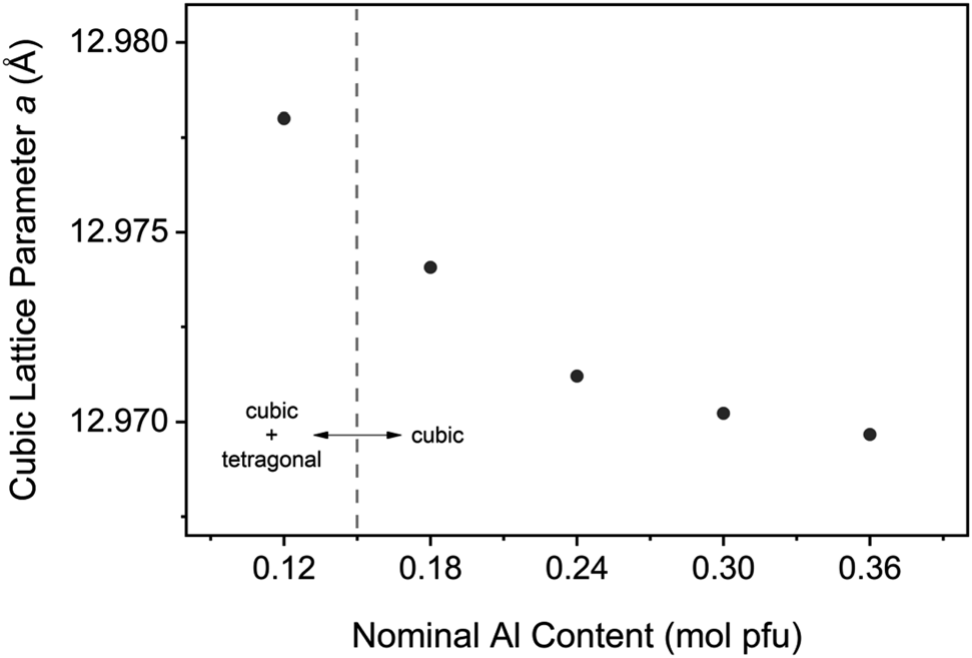
Cubic Al-LLZO lattice parameter variation with aluminium content for samples synthesized by SB technique.

To provide structural evidence of Al incorporation into the cubic lattice, LLZO was synthesized with varying dopant contents and changes in the cell parameter were examined (see Fig. 13). An undoped sample was also synthesized for reference and included in the analysis. Bulk composition was measured by ICP-OES to confirm the Al content in each sample, with the results showing excellent agreement between nominal and measured values (see Table S1†). Rietveld refinements of XRD data were conducted to model the lattice parameter for both the cubic and tetragonal LLZO phases. The results of the refinement are shown in Table 1. A negative trend is observed for the cubic cell parameter *a* with increasing Al content, indicating that the aluminium is being substituted into the garnet framework. This trend is consistent with literature findings that propose Al substitution occurs at the Li site,^26^ since the ionic radii of tetrahedrally coordinated Al^3+^ (0.39 Å) is smaller than that of Li^+^ (0.59 Å).^27^ Another notable feature from Fig. 13 is the decreasing change in the lattice parameter as the Al concentration is increased, indicating a deviation from Vegard's Law which states that the cell parameter varies linearly as a function of composition.^28^ The reduced structural variation observed for the over-doped samples (>0.24 mol Al pfu) is attributed to the formation of secondary phases such as LaAlO_3_, as detected in the XRD measurements (see Fig. S2†). Therefore, there is a critical Al concentration which, if exceeded, will allow lanthanum aluminate impurities to precipitate.

**Table 1:** Results of Rietveld refinement of XRD data for varied Al content

Nominal Al content (mol pfu)	0	0.12	0.18	0.24	0.30	0.36
*R*_wp_ (%)	5.73	6.03	5.47	5.91	5.93	5.81
*R*_p_ (%)	4.45	4.56	4.27	4.59	4.61	4.57
*R*_exp_ (%)	3.59	3.59	3.6	3.77	3.77	3.78
*χ* ^2^	1.59	1.68	1.52	1.57	1.57	1.54
Cubic structure						
Space group		*Ia*3̄*d* (no. 230)				
*R*_Bragg_		2.547	2.975	3.19	3.459	3.436
Cell parameter *a* (Å)		12.97800(19)	12.97407(4)	12.97121(4)	12.97022(4)	12.96967(4)
Tetragonal structure						
Space group	*I*4_1_/*acd* (no 142)					
*R*_Bragg_	3.27	1.976				
Cell parameter *a* (Å)	13.12204(7)	13.0556(3)				
Cell parameter *c* (Å)	12.66879(8)	12.8028(5)				

It should be noted that while the calculated LLZO phase fraction was pure cubic at Al concentrations ≥0.18 mol pfu, it is possible that there is a proportion of the tetragonal phase that was not detected in the XRD measurements. The difficulty of detecting tetragonal LLZO in sub-critically doped garnets by XRD has been reported previously in the literature, and other techniques such as NPD, SEM, and Raman spectroscopy have been used to confirm its presence in the material.^29^

## Discussion

According to the results described above, the phase-purity and crystal structure of the LLZO product were directly impacted by the elemental homogeneity in the precursor mixture, as discussed below.

### Role of precursor homogeneity

Conventional SS synthesis approaches were shown to exhibit significant inhomogeneities with segregated clusters of reactant species observed in the EDS mapping of the starting materials. Given that the stabilization of the preferred cubic phase requires homogenous substitution of Al into the crystal structure, it is unsurprising that the XRD results for SS samples showed residual peaks for the tetragonal LLZO structure. *In situ* analyses further demonstrated the importance of ensuring that the dopant species are locally available for substitution into the crystal structure in maintaining the cubic symmetry on cooling. Additionally, poorly distributed starting materials can lead to the formation of other unwanted secondary phases such as LaAlO_3_, which also appeared in the diffraction patterns for the SS calcined and sintered products. It should be noted that a typical SS synthesis approach would employ multiple grinding and heat treatment steps to eliminate these secondary phases and obtain a high-purity product. However, such strategies introduce additional time and energy requirements which have significant implications on the viability of scaled-up production. Hence, the atomic-level mixing achieved in the SB process was effective in homogeneously distributing the reactant species to achieve and maintain phase-purity through a single-step calcination process. In addition, the SB synthesis route enabled fine-tuning of the precursor stoichiometry to compensate for losses from volatilization to obtain a single-phase material. Ensuring an even distribution of reactants in the precursor is therefore necessary in maintaining control over the phase purity of the product.

Another advantage of enhanced precursor homogeneity was the reduction in the formation temperature of the cubic LLZO phase. The early formation in the SB sample was a result of the minimized diffusion lengths afforded by improved homogeneity between reactants and by the avoidance of subsequent partial melting events during heat treatment. In turn, this accelerated the kinetics of the reaction which is primarily limited by mass-transport. In addition to reduced energy requirements and shorter processing times, greater control over the grain size and morphology can also be obtained with lower operating temperatures. Retaining the nanostructure of the material is an important feature in obtaining high-density ceramic products during sintering.^30^ As demonstrated in Fig. 8, the material prepared by solution chemistry showed improved sintering behavior and maintained phase-purity after densification. Therefore, enhanced homogeneity and particle size control were critical factors in the sintering and densification process. The minimized diffusion lengths between reactants may also provide advantages for emerging bulk SSE production methods, such as the Ultrafast High-Temperature Sintering (UHS) process which benefits from intimate particle contact in the green body compact.^31^

### Product homogeneity

Important surface *vs.* bulk effects are revealed by comparing the phase formation from *in situ* XRD and NPD, suggesting that the effective gas atmosphere plays a critical role during synthesis by directly influencing the formation temperature of LLZO. Specifically, considering LLZO is an oxidized phase and CO_2_ is a product of the garnet formation reaction,^17^ the synthesis kinetics can be accelerated by increasing the oxygen partial pressure and reducing the CO_2_ partial pressure that comes from carbonate decomposition. With this hypothesis, the surface layer with faster gas exchange would react earlier than the internal bulk material. This effect is amplified with compressed pellets and large bulk volume where internal gas exchange is limited, thereby retarding the garnet synthesis reaction. The bulk reaction would be further delayed in the SB material due to the significant release of gaseous carbon during calcination (see DSC results in Fig. 9), promoting a greater CO_2_ partial pressure in the sample environment. This theory is validated by comparing the formation temperatures of LLZO from *in situ* XRD and NPD, as XRD is surface-sensitive while NPD measures the bulk average. The observed disparity of the phase evolution from the surface *vs.* bulk offers some guidance on the synthesis of LLZO. This is significant since compressed pellets are often used over powders for conventional SS synthesis to enhance physical contact for diffusion. However, these findings suggest that the use of compressed pellets may not be necessary for the synthesis of LLZO when the precursor material is already highly homogeneous, as obtained by the SB preparation method.

Additional details about the homogeneity of the products can be obtained by analyzing the peak profiles in the X-ray and neutron diffraction patterns. An apparent broadening effect was observed for the SS sample peaks across both ambient and *in situ* XRD measurements. Considering the inverse relationship between crystallite size and peak width described by the Scherrer equation,^32^ the crystallite size of the SS sample would be expected to be smaller than that of the SB sample. As determined by HRTEM analysis, the SB sample exhibited a larger and more uniform crystallite size (0.5–1 μm) compared to that of the SS material. The rounded morphology of the SB domains is indicative of early-stage densification *via* crystallite coalescence into larger single-crystalline entities, which is further evidenced by the partial necking observed in the SEM images. The onset temperature of the densification process is reduced in the SB material due to the lower formation temperature of the cubic LLZO phase, as shown in the *in situ* XRD analysis. In contrast to the rounded SB sample morphology, the SS sample is comprised of multi-faceted agglomerates of 30–500 nm crystallites. Given the sensitivity of the LLZO system to minor variations in composition and dopant content, local variations of the phase chemistry and lattice parameter may give rise to grain-boundary pinning effects in the material. Such phenomena occur when defects such as secondary impurities or lattice distortions hinder the migration of grain boundaries and increase the activation energy required for grain growth.^33,34^ As shown, diffraction peak profiles and direct observations of the microstructure can be correlated to demonstrate the enhanced homogeneity and, by extension, the improved sinterability of the material prepared by SB processing.

In addition to the effects of crystallite size, increased peak width may also be an indication of chemical inhomogeneity in the sample, whereby a distribution of lattice spacings under the same peak contributes to the broadening of the profile.^35^ As the peak area remains constant, a corresponding reduction in peak intensity is also observed. This effect was consistent across ambient and *in situ* XRD measurements at RT, which showed lower intensities for the SS sample along with a shouldering effect on the cubic peaks which were attributed to phase inhomogeneity due to the presence of tetragonal LLZO. Even at high temperature when the cubic modification is the stable phase (>600 °C), an increased intensity of the Al-LLZO peaks was observed in the SB sample by both XRD and NPD *in situ* measurements. Since local Al content directly influences the cell parameter of cubic LLZO (see Fig. 13), the peak intensities at high temperature are an indication of the spatial distribution of the dopant species in the material. Therefore, by tailoring the synthesis route to enhance dopant homogenization, greater control over the crystallinity and chemical homogeneity of the final cubic phase can be attained.

Control over the dopant incorporation into the cubic structure was demonstrated by monitoring the cell parameter variation with Al content. A reduction in the lattice parameter was observed with increasing Al content, providing evidence of Al substitution into the cubic framework. This analysis was conducted using the SB synthesis technique to enhance the spatial distribution of the doping element and reduce the effects of local inhomogeneities. A significant finding from this analysis was the boundary at which the LLZO structure became purely cubic, above which there was no detection of the tetragonal phase by XRD measurements. This transition occurred between 0.12 and 0.18 mol Al pfu, which is lower than typical values reported in the literature that suggest a concentration greater than 0.204 mol Al pfu is required to stabilize the cubic phase.^26,36^ The observed solubility limit for the LaAlO_3_ impurity is also reduced though SB processing to the 0.24–0.3 mol Al pfu range, with peaks appearing in the XRD pattern at 0.3 mol Al pfu (Fig. S2e†). Studies from Sakamoto *et al.* and Kumazaki *et al.* on Al-LLZO synthesized by SS processing showed evidence of LaAlO_3_ formation at 0.389 and 0.40 mol Al pfu, respectively.^36,37^ Therefore, the optimal dopant content is reduced through the SB synthesis technique in comparison to previous compositional studies that have used SS processing as a point of reference. This is because the SB preparation technique, unlike conventional ceramic processing methods, ensures that the dopant species are fully homogenized in the starting mixture. These findings suggest that the measurement of certain intrinsic properties (phase boundaries, dopant solubility limits *etc.*) can be influenced by the preparation method and its associated effects on elemental homogeneity. Further, it has been shown that the Li vacancy density for active octahedral sites in cubic LLZO are maximized at the phase boundary of cubic and tetragonal LLZO.^19^ Considering that the Li vacancy density is directly correlated to ionic conductivity, an accurate definition of the intrinsic cubic-to-tetragonal phase boundary is critical to optimize LLZO performance. Hence, the presence of local inhomogeneities often arising from SS processing can, in some cases, obscure the accurate determination of certain fundamental properties that dictate electrolytic performance (*i.e.*, optimal doping level).

### Implications for the processing of solid electrolytes

Despite the known problems with material homogeneity and processibility, the research community continues to extensively use conventional SS methods to study the fundamental properties of solid electrolytes. Moreover, as the commercial application of solid electrolytes begins to materialize, the need for alternative scalable and low-cost synthesis methods becomes increasingly important. This is because conventional SS preparation methods, which largely dominated the literature on LLZO until recent years, require high processing temperatures and lack the flexibility to tailor grain sizes down to the nanoscale. The volatilization of Li at high temperatures presents a significant issue in the processing of LLZO due to the sensitivity of the structure and phase chemistry to Li concentration, which in turn affects the ionic conductivity.^36^ This phenomenon also impacts the reproducibility of the process since the off-stoichiometry introduced during heat treatment is difficult to control and can vary considerably between batches. In response to these issues, many research efforts have focused on the development of alternative synthesis routes that enable the formation of LLZO with the desired structure and composition at lower temperatures and shorter processing times. In addition to decreased energy demand and equipment degradation, reduced processing temperatures also enable smaller particle sizes which facilitate further processing into dense, thin membranes necessary for practical ASSBs.

Here, a facile and scalable approach is demonstrated to synthesize pure cubic-LLZO at reduced temperatures with tunable structural parameters and dopant stoichiometry. Moreover, the present analysis goes beyond the synthesis method itself to investigate why the homogeneity at the precursor stage is critical to reduce processing temperatures and maintain control over the morphology, phase chemistry, and crystal structure of the final material. Both spatial and structural evidence is provided to demonstrate how the enhancement of precursor homogeneity can accelerate reaction kinetics to reduce the processing requirements of LLZO. Inhomogeneities from SS processing were preserved even after further heat treatment, while pellets prepared by the SB method showed improved sintering behavior and retained uniform composition and structure. The results provide guidance to optimize existing techniques and may also facilitate the development of novel synthesis strategies where fine-tune control over the final physical properties is required for high-performance applications.

## Conclusions

Control over the crystal structure and phase-evolution of Al-doped LLZO has been demonstrated by manipulating precursor homogeneity and using compensating additions for losses of volatile components. *In situ* XRD and NPD investigations coupled with SEM/EDS observations reveal a strong correlation between the spatial distribution of the doping element and the stability of the cubic LLZO phase. Here, a solution-based synthesis technique is employed to introduce atomic-level mixing and enhance dopant homogeneity to mitigate the formation of the low-conductivity tetragonal phase. Further, the observed increase in cubic-LLZO obtained through the solution-based approach persisted without phase transformation upon cooling, which is an essential requirement for the application of this material as an SSE. Comparatively, conventional solid-state processing showed significant formation of the unwanted tetragonal LLZO modification. Impurity phases were also eliminated by improving elemental homogeneity in the starting mixture, along with a reduction in the formation temperature of the cubic-LLZO phase to 700 °C as observed by *in situ* XRD. Moreover, to account for volatilization during SB condensate formation, the excess addition of Zr-precursor could be fine-tuned to reproducibly obtain a single-phase cubic-LLZO product.

Through this work, precise control over material characteristics is demonstrated by adjusting various synthesis-related parameters. It is expected that the experimental techniques presented here can be applied to other solid electrolyte systems to enable the reproducible synthesis of high-performance materials for future ASSBs.

## Conflicts of interest

There are no conflicts of interest to declare.

## Supplementary Material

RA-012-D2RA03303H-s001
