# Supplementary material for: Compositional and structural control in LLZO solid electrolytes
Source: RSC Adv. 2022 Aug 17;12(36):23466–80. doi: 10.1039/d2ra03303h (PMC9382651; doi:10.1039/d2ra03303h)
Supplement: RA-012-D2RA03303H-s001 [file RA-012-D2RA03303H-s001.pdf]

Electronic Supplementary Material (ESI) for RSC Advances.
This journal is © The Royal Society of Chemistry 2022

Supporting Information

Compositional and structural control in LLZO solid electrolytes

Kade Parascos[1], Joshua L. Watts[1], Jose A. Alarco[2], Yan Chen[3] and Peter C. Talbot[1]*

[1] National Battery Testing Centre, Queensland University of Technology, Brisbane, QLD 4001, Australia

[2] Centre for Clean Energy Technologies and Practices, Centre for Materials Science, Queensland University of Technology, Brisbane 4001, Australia

[3] Neutron Scattering Division, Oak Ridge National Laboratory, Oak Ridge, TN 37831, USA

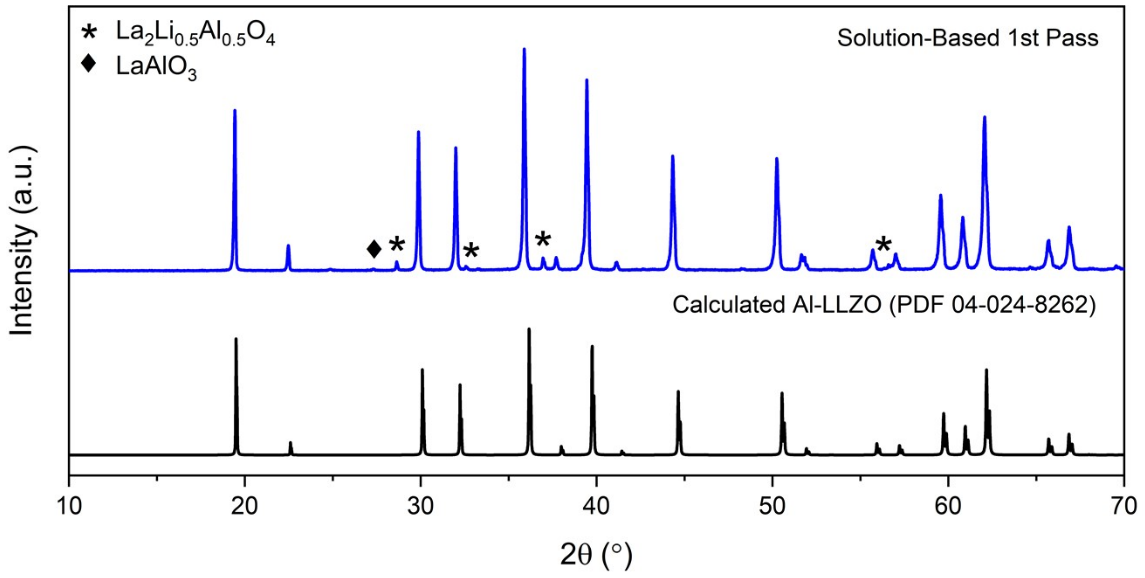

Figure S1. XRD pattern for calcined Al-LLZO powders for the SB sample with 0wt% excess Zr-precursor. The theoretical diffraction pattern of cubic Al-LLZO is included for reference.

Table S1. ICP-OES data for Al-LLZO samples with varying dopant contents where nominal stoichiometry was based on $Li_{7-3x}Al_xLa_3Zr_2O_{12}$. Data has been normalized to La. Chemical formulae have been calculated from molar ratios and oxygen content has been estimated from charge balance calculations.

Nominal Al Content *x* (mol pfu)	Measured Al Content *x* (mol pfu)	Nominal Al wt%	Measured Al wt%	Li/La	Al/La	Zr/La	Chemical Formula
**0**	0	0	0	2.69	0	0.54	$Li_{8.08}La_3Zr_{1.62}O_{11.78}$
**0.12**	0.12	0.39	0.38	2.53	0.04	0.60	n/a†
**0.18**	0.18	0.58	0.59	2.47	0.06	0.61	$Li_{7.41}Al_{0.18}La_3Zr_{1.84}O_{12.16}$
**0.24**	0.25	0.77	0.80	2.39	0.08	0.63	$Li_{7.16}Al_{0.25}La_3Zr_{1.89}O_{12.23}$
**0.3**	0.32	0.96	1.02	2.37	0.11	0.63	n/a††
**0.36**	0.38	1.15	1.22	2.28	0.13	0.60	n/a††

†Composite of cubic and tetragonal LLZO phases
††Impurities $LaAlO_3$ and $La_2Zr_2O_7$ present (see Figure S2)

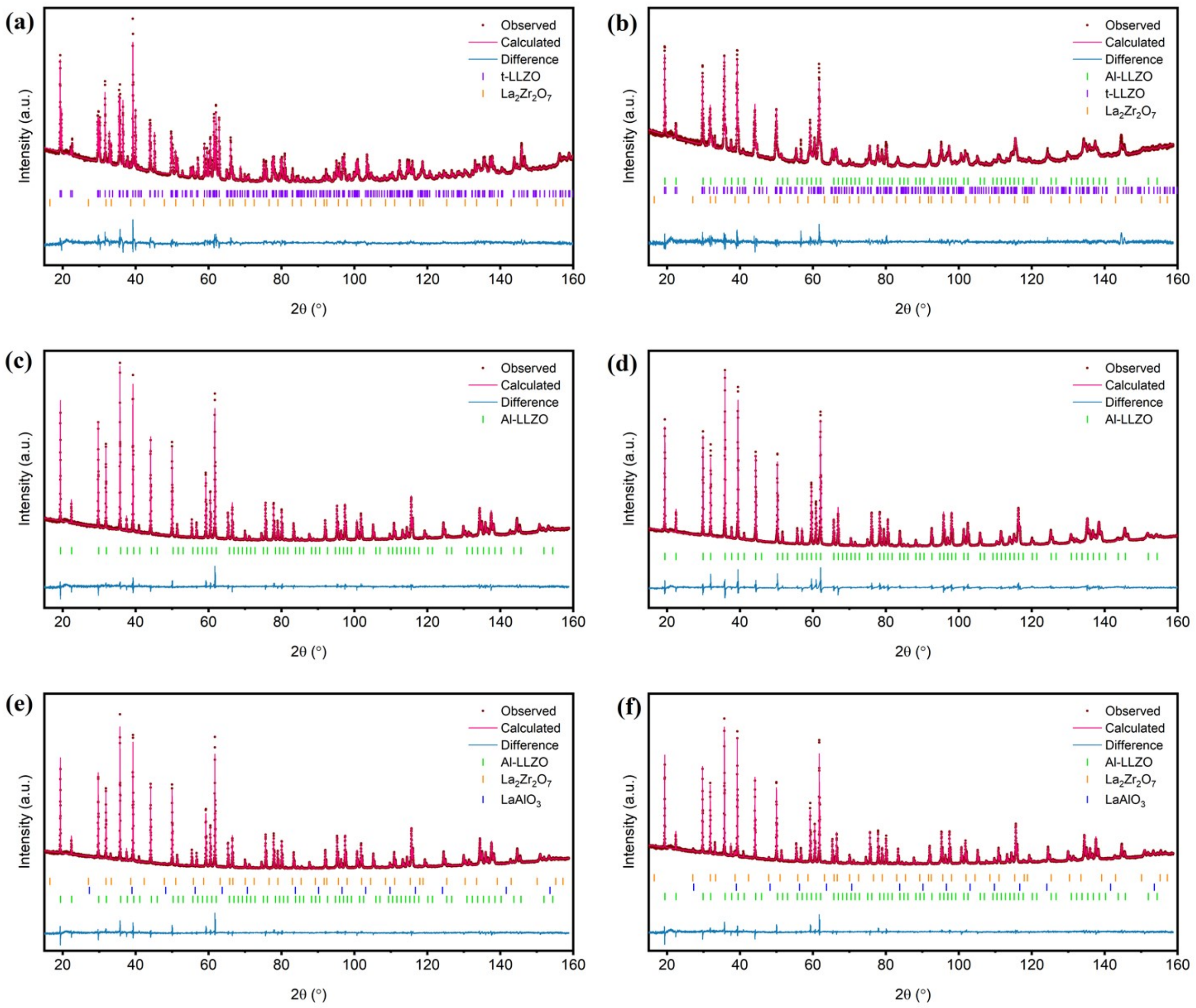

Figure S2. Rietveld refinement plots for calcined LLZO samples prepared by the SB method with an Al content (mol pfu) of (a) 0, (b) 0.12, (c) 0.18, (d) 0.24, (e) 0.3 and (f) 0.36.
